# Standardised Models for Inducing Experimental Peritoneal Adhesions in Female Rats

**DOI:** 10.1155/2014/435056

**Published:** 2014-04-08

**Authors:** Bernhard Kraemer, Christian Wallwiener, Taufiek K. Rajab, Christoph Brochhausen, Markus Wallwiener, Ralf Rothmund

**Affiliations:** ^1^University Hospital for Women, University of Tuebingen, Calwerstraße 4, 72076 Tuebingen, Germany; ^2^Brigham & Women's Hospital, Harvard Medical School, Boston, MA 02115, USA; ^3^Department of Pathology, University Hospital Mainz, Langenbeckstraße 1, 69120 Heidelberg, Germany; ^4^University Hospital for Women, Im Neuenheimer Feld 440, 69120 Heidelberg, Germany

## Abstract

Animal models for adhesion induction are heterogeneous and often poorly described. We compare and discuss different models to induce peritoneal adhesions in a randomized, experimental in vivo animal study with 72 female Wistar rats. Six different standardized techniques for peritoneal trauma were used: brushing of peritoneal sidewall and uterine horns (group 1), brushing of parietal peritoneum only (group 2), sharp excision of parietal peritoneum closed with interrupted sutures (group 3), ischemic buttons by grasping the parietal peritoneum and ligating the base with Vicryl suture (group 4), bipolar electrocoagulation of the peritoneum (group 5), and traumatisation by electrocoagulation followed by closure of the resulting peritoneal defect using Vicryl sutures (group 6). Upon second look, there were significant differences in the adhesion incidence between the groups (*P* < 0.01). Analysis of the fraction of adhesions showed that groups 2 (0%) and 5 (4%) were significantly less than the other groups (*P* < 0.01). Furthermore, group 6 (69%) was significantly higher than group 1 (48%) (*P* < 0.05) and group 4 (47%) (*P* < 0.05). There was no difference between group 3 (60%) and group 6 (*P* = 0.2). From a clinical viewpoint, comparison of different electrocoagulation modes and pharmaceutical adhesion barriers is possible with standardised models.

## 1. Introduction

Adhesions are a serious problem in operative gynaecology [1, 2] and have become the most common complication of abdominal surgery [3]. A number of animal models have already been established to study postoperative adhesions [4]. The cascade that eventually leads to adhesion formation after peritoneal trauma is complex, and inflammatory substances as well as receptors and messengers have been identified that seem to play a substantial role [5–7]. However, these experimental models for adhesion induction are heterogeneous and sometimes lack detailed description, which makes it difficult to correctly reproduce the presented methods or to compare the results or the pathophysiological consequences. Consequently, direct comparison of the results reported in the literature for different adhesion prevention strategies is difficult, and the optimal adhesion barrier for human patients has not yet been found [8, 9].

In the present report, we compare and discuss different models to induce peritoneal adhesions in *n* = 72 rats, using various modalities to traumatize the peritoneum. We also established an objective, standardised, and easily reproducible model for inducing postoperative adhesions consistently in all rats using electrocoagulation. The results were verified by a second look after 14 days. Using the animal model, we intend to mimic the surgical modalities applied in female patients. It serves as a reliable basis for further studies on adhesion formation and prophylaxis, as different barriers can easily be compared [8, 9]. This is a demanding issue for all surgical disciplines, as the superiority of one specific adhesion barrier over other products on the market has not yet been proven. In addition, it is still unclear whether site-specific or nonsite-specific barriers should be applied in clinical routine, which highlights the relevance of animal models which allow significant comparison.

## 2. Animals, Material, and Methods

### 2.1. Study Design

This was a prospective, randomized, and exploratory animal study. A total of 72 rats were randomized into 6 treatment groups before undergoing traumatisation of the peritoneum. The study protocol was approved in advance by the Ethics Committee of the Medical Faculty of the University of Tuebingen, Germany, as project number F 1/06.

### 2.2. Animals

Female, virgin Wistar rats (Crl:WI, Charles River Laboratories, Sulzfeld, Germany) (*n* = 72) with a weight range of 220–280 g were housed for a minimum of 4 days before surgery under standardized laboratory conditions (temperature 21°C ± 2°C, humidity 55% ± 10%, 12 : 12-hour light-dark-cycle). Food (10 mm pellets, Provimi Kliba AG, Kaiseraugst, Switzerland) and tap water was available ad libitum. Preoperatively, six animals were kept per cage (1354G Eurostandard Type IV cages, Tecniplast Deutschland Gmbh, Hohenpeissenberg, Germany) in no particular order. Cages were lined with 5 × 5 × 1 mm wood chips (Abedd - lab & vet Service GmbH, Vienna, Austria). Following surgery, the animals were housed in separate cages (1291H Eurostandard Type III H cages, Tecniplast Deutschland GmbH, Hohenpeissenberg, Germany) until postoperative day two to prevent cannibalisation of the laparotomy wounds. These cages were lined with unbleached chemical pulp (Paul Hartmann AG, Heidenheim, Germany). After postoperative day two, six animals were kept per cage (1354G Eurostandard Type IV cages, Tecniplast Deutschland GmbH, Hohenpeissenberg, Germany) in no particular order to allow social interaction of the animals. These cages were lined with 5 × 5 × 1 mm wood chips (Abedd - lab & vet Service GmbH, Vienna, Austria). No special postoperative nutrition was offered. The husbandry of the animals was in keeping with the European standard requirements.

### 2.3. Interventions

The surgical procedures were performed under aseptic conditions in a dedicated microsurgical animal operating theatre.

**Figure 1:**
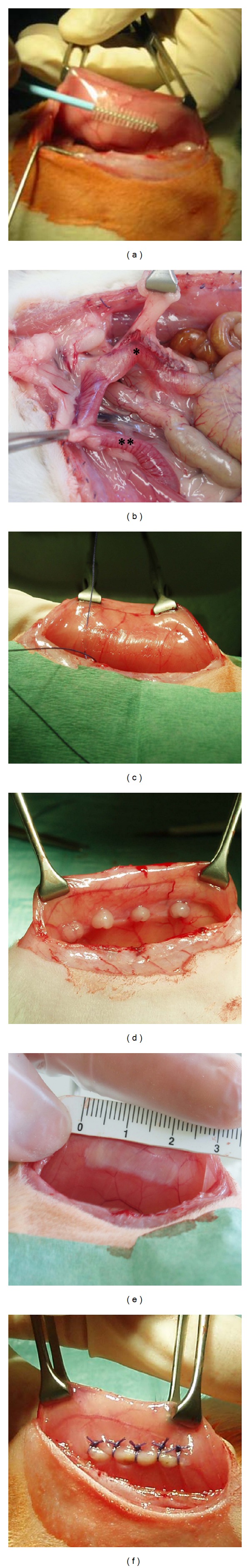
Different modalities to induce peritoneal trauma (a)–(f): (a) and (b) groups 1 and 2, ∗ right uterine horn, ∗∗ left uterine horn, (c) group 3, (d) group 4, (e) group 5, and (f) group 6.

Anaesthesia was induced by inhaling nebulised Isoflurane (Abbott, Wiesbaden, Germany) with the animals breathing spontaneously. Analgesia was provided using preoperative subcutaneous injection of buprenorphine (0.05 mg/kg). The operations were performed under aseptic conditions with the animals on heat mats (ThermoLux Waermeunterlage, Witte + Sutor GmbH, Murrhardt, Germany) warmed to 38°C. After shaving the ventral area with electrical clippers (Favorita II, Aesculap AG, Tuttlingen, Germany), the surgical field was disinfected (Softasept N, B Braun, Melsungen, Germany). Sterile covers (Cardinal Health, Voisins le Bretonneux, France) were fenestrated and applied to the surgical field. The operations were limited to <20 minutes for each rat to minimize the effect of room air tissue drying and all operations were performed by the same surgeons (BK, TR) using powder-free gloves. After ventral midline incision of the skin over a length of 4 cm, the musculoperitoneal layer was incised with a scalpel and opened using surgical scissors over a length of 5 cm at the linea alba. The intraperitoneal cavity was exposed with 2 hooks and handling of the bowel was avoided. Each animal was then allocated into a treatment group using a randomization plan. According to the treatment groups 1–6, the parietal peritoneum was subsequently traumatised with various modalities (Figures 1(a)–1(f)).

#### 2.3.1. Group 1: Visceral & Parietal Brushing (*n* = 12)

Adhesions were induced according to a standardised and validated model established by our group and described in detail in a previous study [10, 11]. Specifically, standardized surgical injuries were applied to both the parietal and visceral peritoneum. A 2.0 × 2.5 cm area of the right and left sidewall peritoneum was brushed with a cytobrush (Gynobrush, Langenbrink, Emmendingen, Germany) (Figure 1(a)) until punctuate bleeding occurred (21 ± 3 strokes) as visual indicator for peritoneal trauma. The same trauma was applied to the uterine horns (Figure 1(b)).

#### 2.3.2. Group 2: Parietal Brushing Only (*n* = 12)

In contrast to group 1, only the parietal peritoneum was brushed. A rectangular 2.0 × 2.5 cm area of the right and left sidewall peritoneum was brushed until punctuate bleeding occurred after 20 ± 2 strokes (Figure 1(a)).

#### 2.3.3. Group 3: Mechanical Denuding and Sutures (*n* = 12)

Adhesions were induced according to a previously published model [12]. Specifically, an area of 2 × 0.5 cm of the parietal peritoneum of the abdominal side walls was excised using a scalpel and forceps. Subsequently the peritoneal defect was closed using five interrupted sutures (4/0 Vicryl, Ethicon, Somerville, NJ) placed equidistantly (5 mm) over the defect with one stitch at the proximal and one stitch at the distal end of the wound. All stitches were made 1 mm from the wound edge (Figure 1(c)).

#### 2.3.4. Group 4: Ischemic Buttons (*n* = 12)

Adhesions were induced according to a previously published model [5, 6]. Four ischemic buttons were created in the parietal peritoneum on one side of the abdomen by grasping a 5 mm button of parietal peritoneum with forceps and ligating the base of the segment with a 4/0 Vicryl suture (Ethicon, Somerville, NJ). The buttons were spaced 1 cm apart along the paracolic gutter (Figure 1(d)).

#### 2.3.5. Group 5: Bipolar Electrocoagulation (*n* = 12)

Adhesions were induced using bipolar electrocoagulation (Vio 300D bipolar generator ERBE Elektromedizin, Tuebingen, Germany). Standardized lesions were inflicted on an area of 0.5 cm × 2 cm by sweeping bipolar electrocoagulation forceps (ERBE Elektromedizin, Tuebingen, Germany) over the abdominal peritoneum for 3 seconds. The traumatised area was 1.5 cm dorsal to the midline incision and centered at the second pair of nipples. The current was delivered using the following settings: Bipolar Soft, Effect 4, 40 Watts. The soft coagulation delivers a sinusoidal current >200 V and ensures a slow and deep haemostasis without adhering to the tissue (Figure 1(e)).

#### 2.3.6. Group 6: Electrocautery and Sutures (*n* = 12)

In this model, we established the combination of standardised electrocautery and sutures for adhesion induction. Traumatisation by electrocoagulation occurred as for group 5. However, the defects were subsequently closed using five interrupted sutures (4/0 Vicryl, Ethicon, Sommerville, NJ) to induce an ischemic field around the traumatised area. The sutures were placed equidistantly (5 mm) over the defect with one stitch at the proximal and one stitch at the distal end of the wound. All stitches were made 1 mm from the wound edge (Figure 1(f)).

In all groups 1–6, the midline incision was closed in two layers with continuous 3/0 Vicryl (Ethicon, Sommerville, NJ) in the musculoperitoneal layer and continuous 3/0 Vicryl in the intracuticular layer. The proximal and distal knots of the intracuticular suture were protected with one additional head seam each. All animals received buprenorphine (0.05–0.1 mg/kg) postoperatively and every 6 hours over the following two days for analgesia. The animals were observed twice daily for signs of wound infection, dehiscence, or other complications. Additional interruption criteria were a lack of social interaction, refusal to eat, and shaggy fur. Animals that were excluded (*n* = 5) were replaced. After 14 days, a second-look laparotomy was performed to assess adhesion formation according to the scoring systems described below. For this purpose, the animals were sacrificed using CO_2_.

### 2.4. Adhesion Evaluation

**Figure 2:**
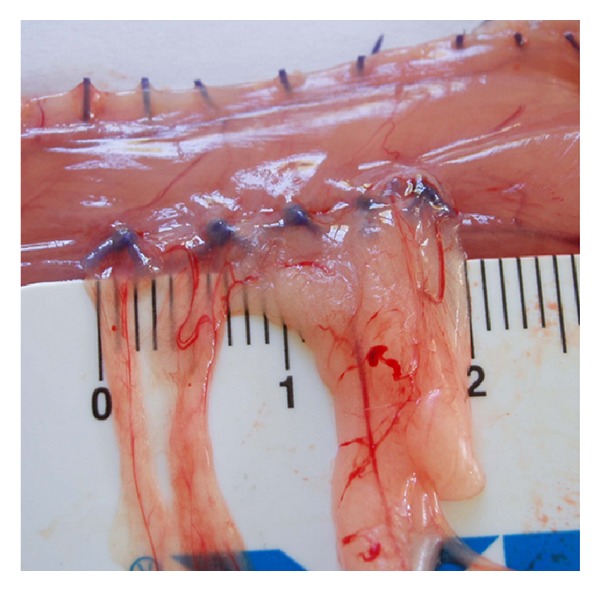
The completely excised abdominal side wall for adhesion scoring.

All adhesions were evaluated by second-look operation and immediately documented with digital photography in a standardised fashion (Olympus Mju Mini digital camera, Olympus, Tokyo, Japan) to be presented to an independent surgeon. The traumatised abdominal sidewall that the adhesions were attached to was excised completely (Figure 2). We assessed quantity (a), quality (b), and histological features (c) of all adhesions that were induced by the different models.

*(a) Quantity.* The quantity was scored according to the incidence of traumatised areas with adhesions and the adhesion coverage was calculated as fraction of adhesions (given as percentage of the traumatised area). 

*(b) Quality.* The quality of the induced adhesions was determined according to the following scores: 0 (no adhesion), 1 (avascular adhesion), 2 (filmy vascular adhesion), and 3 (dense, vascular adhesion). Adhesion quality was considered “filmy” if the scale of a ruler was visible through the tissue; otherwise, it was considered “dense” (Figure 2). 

*(c) Histology.* All specimens were evaluated histologically. The adhesive fibrous tissue was dissected with the continuity of the transition zone to the macroscopic normal peritoneal wall. All specimens were fixed in buffered formalin (4%) and embedded in paraffin according to standardized methods. For histological evaluation, hematoxylin eosin staining was performed automatically with a Leica-stainer (ST4040, Leica, Germany) using standardized methods. Staining of fibrous tissue using Elastica van Gieson and Goldner staining, as well as fibrin staining using staining according to Pears was performed according to standard laboratory methods.

### 2.5. Statistics

Differences between groups in* adhesion coverage* were analysed using pairwise Wilcoxon tests with Bonferroni correction for multiple testing. Since ties are present, the exact version of the test from the package “exactRankTests” of the statistics software R (R Foundation for Statistical Computing, Version 2.12.1) was used. The overall significance level was set to 0.05.

The different categories of* adhesion quality* are presented descriptively and were not tested with a statistical test to avoid multiple testing of combined characteristics.

## 3. Results

In total, 5 animals were excluded due to anaesthetic complications (*n* = 2), uncontrollable sepsis (*n* = 1), and bowel evisceration (*n* = 2) after autocannibalism.

**Figure 3:**
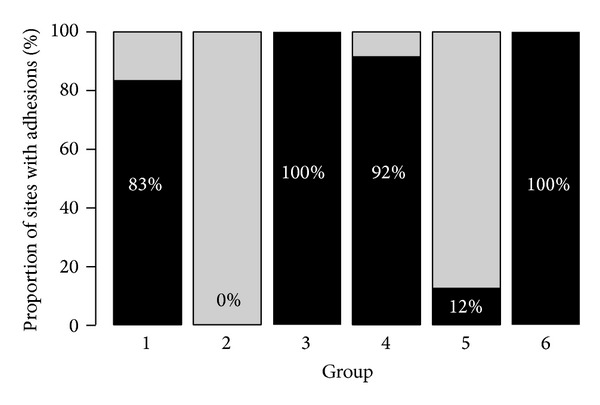
Proportions of sites with adhesions in the groups 1–6.

In group 1,* parietal and visceral brushing*, 83% (Figure 3) of traumatised areas were associated with adhesion formation. The proportion of the adhesions of the traumatised area was 48%. 62% of the adhesions were dense, 21% were filmy, and 17% were avascular. All animals survived. Histologically, in the brush model an inhomogeneous picture could be observed, partly with light fibrosis and partly with subserosal inflammation. Multifocal minimal deposits of fibrin could be found. The serosa was covered by a continuous layer of flat mesothelial cells.

In group 2,* parietal brushing only*, no adhesion formation was observed. Again, all animals survived. In cases of adhesion formation, the histological picture was similar to that of group 1.

**Figure 4:**
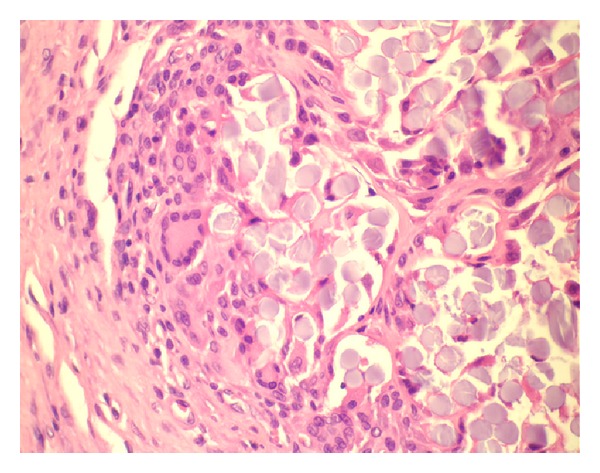
H&E stained slide showing foreign body reaction.

In group 3,* peritoneal denuding combined with suturing*, 100% of traumatised areas were associated with adhesion formation. The proportion of the adhesions of the traumatised area was 61%. 63% of the adhesions were dense, 17% were filmy, and 20% were avascular. One animal was excluded. Histology revealed a mild fibrosis of the serosa covered by resting mesothelial cells. Multifocal areas with residues of fibrin clots with macrophages, inflammatory cells, and foreign body reaction were evident (Figure 4). The muscular layer was not affected.

In group 4,* ischemic button formation*, 92% of traumatised areas were associated with adhesion formation. The proportion of the adhesions of the traumatised area was 71%. 72% of the adhesions were dense and 28% were filmy. Two animals were excluded. Histomorphological, ischemic damage of the muscular tissue of the abdominal wall could be demonstrated combined with a granulocytic infiltrate, a subserosal oedema with huge amounts of fibrin, and a capillary rich tissue with moderate granulocytic infiltrates. Furthermore, a large number of spindle shape cells could be detected. Neither a foreign body reaction nor a significant fibrosis could be detected. The formed adhesions consisted of a mature fat tissue and were covered by a single cell layer of mesothelial cells.

In group 5,* bipolar parietal electrocoagulation*, 12% of traumatised areas were associated with adhesion formation. The proportion of the adhesions of the traumatised area was 4%. All of these adhesions were dense. 1 of the 12 animals died. Histologically, the specimens of the coagulation group showed an inflammatory infiltrate in the serosa, subserosa, and the muscular layers of the abdominal wall. Furthermore, multifocal circumscribed areas of muscle cell necrosis could be found. At the basis of the peritoneal adhesions, the subserosa was rich in small vessels with a mild inflammatory infiltrate.

In group 6,* electrocautery combined with suturing*, all trauma sites were associated with adhesion formation. The proportion of the adhesions of the traumatised area was 66%. 50% of the adhesions were dense, 25% were filmy, and 25% were avascular. One animal was excluded. Histologically, all specimens showed dense adhesive bands covered by flat mesothelial cells. The base of the adhesive bands was in all cases located at the suture.

Figure 3 summarizes the proportions of sites with adhesions in groups 1–6.

### 3.1. Statistical Comparison

Analysis of the adhesion coverage according to pairwise Wilcoxon tests with Bonferroni correction for multiple testing showed that groups 2 and 5 were significantly less than the other groups (*P* < 0.01). Group 6 was significantly higher than group 4 (*P* = 0.032).

## 4. Discussion

Rats are widely established for adhesion research, as the pre- and postoperative management is favourable compared to larger animals [13–15]. As the mesothelial monolayer is extremely delicate and hence susceptible to abrasion even with meticulous surgical technique [16], the traumatisation of the peritoneum for adhesion induction is the key factor that all existing models have in common. The aim of our study was to compare and contrast various rat models and to set up a novel model, traumatising the parietal peritoneum by electrocoagulation (group 6).

Laparotomy with ventral midline incision is a convenient and rapid approach, and as shown before, is not associated with additional adhesion formation [12, 17]. Laparoscopic models exist [18]; however, they require a specialised setting and the pneumoperitoneum may confound adhesion formation [19].

To minimize the number of animals used, a model that reliably leads to adhesion formation in the sacrificed rats is desirable. This can be achieved by a consistently adhesion-inducing trauma and the optimal time for adhesion assessment. The time window for second look depends on the objective of the study, but it is generally accepted that adhesion formation is complete by day 7 [20]. We performed a second look after 14 days in order to achieve complete adhesion formation.

An adequate model is easily reproducible between laboratories in a standardised fashion and leads to the formation of objectively scorable adhesions [21]. The brush models (groups 1 and 2) are quickly practicable; however, the resulting adhesions do not occur in all traumatised animals. It is impossible to optimally standardize the traumatized area, as the applied pressure of the brush is inconsistent over the area and the number of strokes required for induction of bleeding is variable. The brush trauma leads to an increased vascular permeability which leads to the exudation of inflammatory cells and consequently to the formation of adhesions. Blood in the abdominal cavity can therefore be a confounding element to adhesion formation [20].

In groups 3–6, sufficient standardisation can be achieved. In group 3 (mechanical parietal denuding and sutures), the area of peritoneal denuding is exactly determined because a defined area can be excised. However, it is often unavoidable that the sharp incision leads to damage of small vessels in the peritoneal surface with subsequent bleeding. As mentioned above, this can confound adhesion formation. In group 4 (ischemic buttons), there is a defined length of parietal peritoneum where the buttons are placed. In groups 5 and 6 (electrocoagulation and electrocoagulation with sutures), the current can be applied to the targeted area and the immediate change in the appearance of treated tissue allows for visual feedback of an even distribution of injury.

Electrocoagulation with or without additional suturing is used extensively to achieve haemostasis and wound closure in female patients. Therefore, we used this combination in the animal model to achieve a high level of comparability with the operative situation in human surgery. This contrasts with previous studies, which rely on modes of traumatisation that are not usually employed in human surgery (brush, creation of ischemic buttons, and sharp peritoneal denuding). Electrocoagulation also differs from other modalities used in experimental models with regard to the quality of the injury produced [20]. It causes local peritoneal trauma and ischemia by the denaturation of tissue proteins and subsequent sealing of blood vessels.

We assessed the quantity and quality of adhesions as described previously. In group 1 (parietal and visceral brushing), 83% of traumatised areas were associated with adhesion formation. In contrast, in group 2 (parietal brushing only), no adhesion formation was observed. To date, it remains controversial whether traumatisation of the parietal or the visceral peritoneum induces more adhesions, but previous data indicate that the potential to form adhesions is significantly higher in visceral than in parietal peritoneal lesions [11]. The resulting adhesion quantity is significantly different. The fact that the visceral peritoneum was not injured in group 2 (parietal brushing only) could be an explanation for the absence of adhesions, as adhesions are more likely to occur when both contact surfaces of the peritoneum are injured [22].

According to the authors, adhesion formation in group 3 (mechanical denuding and sutures) is not caused by the suture itself, since the suturing of the midline incision, as in our experiment, did not lead to significantly more adhesions. It can be explained instead by the local ischaemia around the traumatised area [12]. However, it has to be stated that sutures have the potential to induce a foreign body reaction with subsequent adhesions [23]. Adhesion formation is increased in combination with ischaemia caused by tight stitches. Bigatti et al. examined neoangiogenesis in adhesion formation and showed a gradual progression in the type and tenacity of adhesion formation in the presence of a silastic patch and ischaemia [24]. Histologically, in contrast to models 5 (bipolar electrocoagulation) and 6 (electrocautery and sutures), the muscular layer of the abdominal wall was not affected in group 3 (mechanical denuding and sutures). However, the sites of the sutures showed circumscribed inflammation and a foreign body reaction.

In group 4 (ischaemic buttons), local ischaemic fields were obtained without additional peritoneal traumatisation according to Reed et al. [5, 6]. Again, we think that in the button model not only ischemia, but also the foreign body reaction caused by the suture plays a role for adhesion formation. This theory can be supported by the comparison of models 5 and 6 (electrocoagulation and electrocoagulation and sutures). In group 5, 88% of the areas traumatised by electrocoagulation only were adhesion free. However, when electrocautery is combined with suturing (group 6), adhesion formation in all treated animals can be observed (Figure 3). This difference is statistically significant.

**Figure 5:**
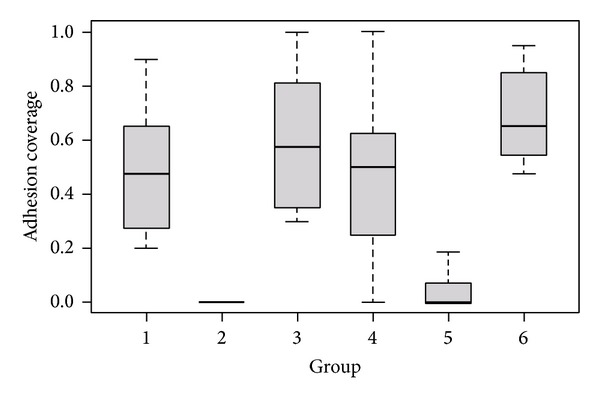
Boxplots: adhesion coverage per group.

The hypothesis of increased adhesion formation after trauma and foreign body is also substantiated when adhesion coverage is assessed in our study. The coverage in the different groups is depicted with boxplots in Figure 5. A series of animals potentially treated with foreign bodies such as untightened suture materials (thread loops) only was not conducted, as none of our *n* = 72 animals showed adhesions located at the midline incisions that always included the parietal peritoneum. This is in concordance with the findings of Holmdahl et al. [12].

Induction models which produce a range of different adhesion qualities are desirable. As shown in our study, the combination of electrocoagulation and suturing (group 6) produced a wider range of different adhesions patterns (50% dense, 25% filmy, and 25% avascular) compared to electrocoagulation alone (group 5) and buttons (group 4). When electrocoagulation is used for haemostasis, it can be expected that different modes and current settings may also affect the outcome of adhesions. To date, from a clinical point of view, it remains unclear how different adhesion qualities (avascular, filmy, and dense) affect patients' symptoms [25]. However, dense adhesions result in prolonged subsequent operations [26], which are also potentially associated with greater risk of enterotomy [27].

A limitation to the presented study is the limited number of animals per group and the lack of follow-up, which is inherent to the study design as a second look was performed after the animals were sacrificed after 14 days. In this study, we took no specific action to keep the tissue moist during the intervention, although tissue desiccation may be one of the factors that leads to adhesion formation. We understand that comparing the results of different traumatized areas, different trauma depths and different causes of tissue trauma will be a complex endeavour; however, this study seeks to present a suitable adhesion animal model and the interpretation of the results as a whole allows for that.

## 5. Summary

A convenient and easily reproducible animal model is pivotal for the further investigation and research of biomedical and surgical issues such as adhesion induction and postoperative formation. Both are highly dependent on trauma and subsequent peritoneal conditions. It is therefore desirable to elaborate models that mimic the clinical situation as closely as possible. Due to the fact that electrocautery is combined with suturing, which reflects the situation in open or laparoscopic surgery, we prefer group 6 for further experimental research. This model combines the application of a defined electrical current (trauma and local inflammation) and suture material (foreign body and local ischemia) for the induction of a wide range of peritoneal adhesions.

## References

[1] Rajab TK, Wallwiener M, Talukdar S, Kraemer B (2009). Adhesion-related complications are common, but rarely discussed in preoperative consent: a multicenter study. *World Journal of Surgery*.

[2] DeWilde RL, Trew G (2007). Postoperative abdominal adhesions and their prevention in gynaecological surgery. Expert consensus position. *Gynecological Surgery*.

[3] de Wilde RL, Brِlmann H, Koninckx PR Prevention of adhesions in gynaecological surgery: the 2012 European field guideline. *Gynecological Surgery*.

[4] Ozel H, Avsar FM, Topaloglu S, Sahin M (2005). Induction and assessment methods used in experimental adhesion studies. *Wound Repair and Regeneration*.

[5] Reed KL, Fruin AB, Bishop-Bartolomei KK (2002). Neurokinin-1 receptor and substance P messenger RNA levels increase during intraabdominal adhesion formation. *Journal of Surgical Research*.

[6] Reed KL, Fruin AB, Gower AC, Stucchi AF, Leeman SE, Becker JM (2004). A neurokinin 1 receptor antagonist decreases postoperative peritoneal adhesion formation and peritoneal fibrinolytic activity. *Proceedings of the National Academy of Sciences of the United States of America*.

[7] Kraemer B, Wallwiener M, Wallwiener CW (2012). Differential mRNA expression of TACR1 after ischemic peritoneal trauma: a pilot animal study. *Archives of Gynecology and Obstetrics*.

[8] Rajab TK, Wallwiener CW, Brochhausen C, Hierlemann H, Kraemer B, Wallwiener M (2009). Adhesion prophylaxis using a copolymer with rationally designed material properties. *Surgery*.

[9] Rajab TK, Wallwiener M, Planck C, Brochhausen C, Kraemer B, Wallwiener CW (2010). A direct comparison of seprafilm, adept, intercoat, and spraygel for adhesion prophylaxis. *Journal of Surgical Research*.

[10] Wallwiener M, Brucker S, Hierlemann H, Brochhausen C, Solomayer E, Wallwiener C (2006). Innovative barriers for peritoneal adhesion prevention: liquid or solid? A rat uterine horn model. *Fertility and Sterility*.

[11] Wallwiener D, Meyer A, Bastert G (1998). Adhesion formation of the parietal and visceral peritoneum: an explanation for the controversy on the use of autologous and alloplastic barriers?. *Fertility and Sterility*.

[12] Holmdahl L, Al-Jabreen M, Risberg B (1994). Experimental models for quantitative studies on adhesion formation in rats and rabbits. *European Surgical Research*.

[13] Hill-West JL, Chowdhury SM, Dunn RC, Hubbell JA (1994). Efficacy of a resorbable hydrogel barrier, oxidized regenerated cellulose, and hyaluronic acid in the prevention of ovarian adhesions in a rabbit model. *Fertility and Sterility*.

[14] Szabo A, Haj M, Waxsman I, Eitan A (2000). Evaluation of Seprafilm and amniotic membrane as adhesion prophylaxis in mesh repair of abdominal wall hernia in rats. *European Surgical Research*.

[15] Bakkum EA, van Blitterswijk CA, Dalmeijer RAJ, Trimbos JB (1994). A semiquantitative rat model for intraperitoneal postoperative adhesion formation. *Gynecologic and Obstetric Investigation*.

[16] DeWilde RL, Trew G (2007). Postoperative abdominal adhesions and their prevention in gynaecological surgery. Expert consensus position, part 2: steps to reduce adhesions. *Gynecological Surgery*.

[17] Moreno A, Aguayo JL, Zambudio G, Ramirez P, Canteras M, Parrilla P (1996). Influence of abdominal incision on the formation of postoperative peritoneal adhesions: an experimental study in rats. *European Journal of Surgery*.

[18] Roman H, Canis M, Kamble M, Botchorishvili R, Pouly J, Mage G (2005). Efficacy of three adhesion-preventing agents in reducing severe peritoneal trauma induced by bipolar coagulation in a laparoscopic rat model. *Fertility and Sterility*.

[19] Molinas CR, Koninckx PR (2000). Hypoxaemia induced by CO_2_ or helium pneumoperitoneum is a co-factor in adhesion formation in rabbits. *Human Reproduction*.

[20] DiZerega GS, Campeau JD (2001). Peritoneal repair and post-surgical adhesion formation. *Human Reproduction Update*.

[21] Kraemer B, Wallwiener M, Petri N (2011). Different approaches for objective scoring of experimental post-operative adhesions in the rat model—a description. *Gynecological Surgery*.

[22] Haney AF, Doty E (1994). The formation of coalescing peritoneal adhesions requires injury to both contacting peritoneal surfaces. *Fertility and Sterility*.

[23] Luijendijk RW, de Lange DCD, Wauters CCAP (1996). Foreign material in postoperative adhesions. *Annals of Surgery*.

[24] Bigatti G, Boeckx W, Gruft L, Segers N, Brosens I (1995). Experimental model for neoangiogenesis in adhesion formation. *Human Reproduction*.

[25] Demco L (2004). Pain mapping of adhesions. *Journal of the American Association of Gynecologic Laparoscopists*.

[26] Coleman MG, McLain AD, Moran BJ (2000). Impact of previous surgery on time taken for incision and division of adhesions during laparotomy. *Diseases of the Colon and Rectum*.

[27] van der Krabben AA, Dijkstra FR, Nieuwenhuijzen M, Reijnen MMPJ, Schaapveld M, van Goor H (2000). Morbidity and mortality of inadvertent enterotomy during adhesiotomy. *British Journal of Surgery*.

